# Quantitative comparison of immunohistochemical staining measured by digital image analysis versus pathologist visual scoring

**DOI:** 10.1186/1746-1596-7-42

**Published:** 2012-06-20

**Authors:** Anthony E Rizzardi, Arthur T Johnson, Rachel Isaksson Vogel, Stefan E Pambuccian, Jonathan Henriksen, Amy PN Skubitz, Gregory J Metzger, Stephen C Schmechel

**Affiliations:** 1 Department of Laboratory Medicine and Pathology, University of Minnesota, 420 Delaware Street SE, MMC76, Minneapolis, MN, 55455, USA; 2 Biostatistics and Bioinformatics Core, Masonic Cancer Center, University of Minnesota, Minneapolis, MN, USA; 3 BioNet, University of Minnesota, Minneapolis, MN, USA; 4 Department of Radiology, University of Minnesota, Minneapolis, MN, USA

**Keywords:** Annotation, Color deconvolution, Digital pathology, Immunohistochemistry, Intensity, Quantification, Software

## Abstract

**Abstract:**

Immunohistochemical (IHC) assays performed on formalin-fixed paraffin-embedded (FFPE) tissue sections traditionally have been semi-quantified by pathologist visual scoring of staining. IHC is useful for validating biomarkers discovered through genomics methods as large clinical repositories of FFPE specimens support the construction of tissue microarrays (TMAs) for high throughput studies. Due to the ubiquitous availability of IHC techniques in clinical laboratories, validated IHC biomarkers may be translated readily into clinical use. However, the method of pathologist semi-quantification is costly, inherently subjective, and produces ordinal rather than continuous variable data. Computer-aided analysis of digitized whole slide images may overcome these limitations. Using TMAs representing 215 ovarian serous carcinoma specimens stained for S100A1, we assessed the degree to which data obtained using computer-aided methods correlated with data obtained by pathologist visual scoring. To evaluate computer-aided image classification, IHC staining within pathologist annotated and software-classified areas of carcinoma were compared for each case. Two metrics for IHC staining were used: the percentage of carcinoma with S100A1 staining (%Pos), and the product of the staining intensity (optical density [OD] of staining) multiplied by the percentage of carcinoma with S100A1 staining (OD*%Pos). A comparison of the IHC staining data obtained from manual annotations and software-derived annotations showed strong agreement, indicating that software efficiently classifies carcinomatous areas within IHC slide images. Comparisons of IHC intensity data derived using pixel analysis software versus pathologist visual scoring demonstrated high Spearman correlations of 0.88 for %Pos (p < 0.0001) and 0.90 for OD*%Pos (p < 0.0001). This study demonstrated that computer-aided methods to classify image areas of interest (e.g., carcinomatous areas of tissue specimens) and quantify IHC staining intensity within those areas can produce highly similar data to visual evaluation by a pathologist.

**Virtual slides:**

The virtual slide(s) for this article can be found here: http://www.diagnosticpathology.diagnomx.eu/vs/1649068103671302

## 

Despite the exceptional utility of genomics methods in the discovery phase of experimentation, these technologies require validation due to problems including misidentification of nucleic acid probes on gene expression microarrays [1,2], non-specificity of probes [3], and the essentially unavoidable false discovery rates associated with massive multiple hypothesis testing [4]. Appropriately powered studies to validate initial results of genomics studies often are lacking [5] or fail to confirm initial discovery-phase results [6], limiting clinical implementation of new disease biomarkers.

Immunohistochemistry (IHC) is an important technique for biomarker validation for several reasons. First, it allows direct visualization of biomarker expression in histologically relevant regions of the examined tissue. This is an important advantage over “grind and bind” assays in which tissue is solubilized for biochemical analysis, which may lead to false negative results if few biomarker-positive cells are present in a background of biomarker-negative tissue elements [7]. Second, clinical laboratories typically perform IHC on FFPE tissue sections processed by standard methods, making potentially available hundreds of millions of specimens for study [8]. Third, validated IHC assays may be implemented readily into clinical practice. For example, genomics methods were used to discover mRNA biomarkers capable of subclassifying diffuse large B cell lymphoma (DLBCL) into prognostically discrete subtypes [9]. Relevant subsets of these gene products were validated at the protein level using IHC on large numbers of DLBCL specimens [10,11], and validated IHC panels are now used clinically.

Traditionally, pathologists have visually scored IHC data. For example, in the calculation of an HSCORE, a summation of the percentage of area stained at each intensity level multiplied by the weighted intensity (e.g., 1, 2, or 3; where 0 is no staining, 1 is weak staining, 2 is moderate staining and 3 is strong staining) of staining is generated [12]. These analyses are frequently performed on specimens arrayed on stained TMA sections allowing representation of a sufficiently large number of specimens to for statistically rigorous testing [13,14]. Tissue specimens are adequately represented by tissue cores on very few slides [15,16] minimizing IHC cost and tissue usage, and facilitating intra-observer, inter-observer and inter-laboratory studies [10,17-20].

Pathologist visual scoring is fraught with problems due to subjectivity in interpretation. Automated IHC measurements promise to overcome these limitations. Whole-slide imaging systems are widely available to convert glass slides into diagnostic quality digital images [21]. Automated IHC measurements are precise in ranges of staining that appear weak to the eye [22] and produce continuous data [23]. Moreover, when automated IHC measurements are provided to a pathologist during visual scoring, computer-aided IHC analysis substantially improves both intra- and inter-observer agreement [20].

In this study, we used TMAs of ovarian serous carcinomas stained with an antibody directed against S100A1 to determine the ability of commercially available software algorithms (Genie Histology Pattern Recognition software suite including Genie Training v1 and Genie Classifier v1, and Color Deconvolution v9, Aperio Technologies, Vista, CA, USA) to replicate results obtained solely through visual inspection by a pathologist. Two specific comparisons were made in this study: a) the segmentation of the digitized tissue images into disease-relevant areas (those containing carcinoma) versus non-relevant areas (stroma and glass) and b) the quantification of stain intensity within areas of carcinoma. Specifically, first computer-derived IHC staining data obtained from both hand-annotated and Genie-classified areas of carcinoma were compared as a measure of agreement in tissue classification. Next, computer-derived IHC staining data from within Genie-classified areas of carcinoma were compared against pathologist visual scores.

## Materials and methods

### TMA Construction, IHC, and Pathologist visual scoring

Four TMA slides representing duplicate 0.6 mm core samples from 215 cases of ovarian serous carcinoma were provided by the Cheryl Brown Ovarian Cancer Outcomes Unit (Vancouver, Canada), stained with primary mouse anti-human S100A1 monoclonal antibody (clone DAK-S100A1/1; DakoCytomation, Glostrup, Denmark), and visualized with 3,3-diaminobenzidine (DAB) as previously described [24]. A total of 54, 54, 77 and 30 cases were represented by TMA 1, TMA 2, TMA 3, and TMA 4, respectively. Each TMA spot was examined by a pathologist (S.E.P.) who assigned a score of 0 (no staining), 1 (<10% of malignant cells staining), 2 (10%-50% of malignant cells staining), or 3 (>50% of malignant cells staining) within carcinomatous areas [24].

### Slide digitization, Manual annotation, and Computer-aided image analysis

Digital images of IHC-stained TMA slides were obtained at 40x magnification (0.0625 μm^2^ per raw image pixel) using a whole slide scanner (ScanScope CS, Aperio) fitted with a 40x/0.75 Plan Apo objective lens (Olympus, Center Valley, PA, USA). Images were saved in SVS format (Aperio), managed with server software (ImageServer, Aperio), and retrieved with a file management web interface (Spectrum, Aperio).

Under pathologist (S.C.S.) supervision, a technician (A.E.R.) hand-annotated tumor regions on whole slide images using Aperio’s annotation software (ImageScope v10, Aperio). For automated image classification, image areas from TMA 1 were annotated that represented three user-defined Image Classes (carcinoma, stroma, and clear glass) and ranged in morphologic appearance and staining intensity of DAB and hematoxylin (counterstain). These image areas were used as input parameters for the histologic pattern recognition training software (Genie Training, Aperio) to produce a Genie Training Set. The effectiveness of the Genie Training Set was visualized on TMA 1 image test regions (TMA spots) using the image classifier algorithm (Genie Classifier, Aperio), which overlaid an image markup pseudocolored for each Image Class. Annotated image areas from TMA 1 were adjusted (adding or removing image areas) for each Image Class to improve the classifier accuracy. For example, if the Genie Classifier algorithm over-classified regions of stroma as carcinoma, additional stromal annotations were added to the Genie Training algorithm to better represent the stromal Image Class. This process of adjusting annotations, re-running the Genie Training algorithm, and visually inspecting pseudocolored markup images output by Genie Classifier was iteratively repeated until a Genie Training Set was developed to classify the TMA 1 slide optimally, as visually validated by a pathologist (S.C.S.). The optimized Genie Classifier was then run on TMAs 1-4.

IHC staining was evaluated within carcinomatous areas of each TMA spot that had been manually annotated, and a separate analysis was performed on areas from each TMA spot that had been classified as carcinoma by the Genie Classifier. As previously described [25,26], the Color Deconvolution algorithm (Aperio) was used to isolate individual stains for quantification: the red, green, and blue (RGB) OD color vectors were measured for each stain using default software settings and control slides stained separately with hematoxylin or DAB. The average RGB OD values (Hematoxylin: 0.682724, 0.642898, 0.347233; DAB: 0.486187, 0.588538, 0.645945) were entered into the Color Deconvolution software to define each stain component in the final analysis settings. Staining was quantified by two metrics: the percentage of carcinoma with S100A1 staining (%Pos), and the product of the staining intensity (OD) multiplied by the percentage of carcinoma with S100A1 staining (OD*%Pos). As previously described, the amount of staining present is linearly related to OD [26].

### Statistical analysis

Duplicate spots were summarized as a single score for each case by randomly selecting one of the replicates. In order to compare pathologist hand and Genie automated annotations, which represent the same clinical measure on the same scale, Bland-Altman plots were used [27]. This scatterplot of the difference between methods, with reference lines at the mean difference and mean difference ± 2*standard deviation of the differences, allows for an assessment of agreement rather than just a measure of correlation. Comparisons of both %Pos and OD*%Pos values by method were conducted. Spearman’s correlation was calculated to compare pathologist visual scores versus %Pos and OD*%Pos values. Each comparison was made within each of the four TMAs. Additionally, we pooled all of the data to compare the %Pos and OD*%Pos values by pathologist score using Wilcoxon rank-sum tests.

## Results

### Hand annotation versus Genie image classification of carcinoma

**Figure 1:**
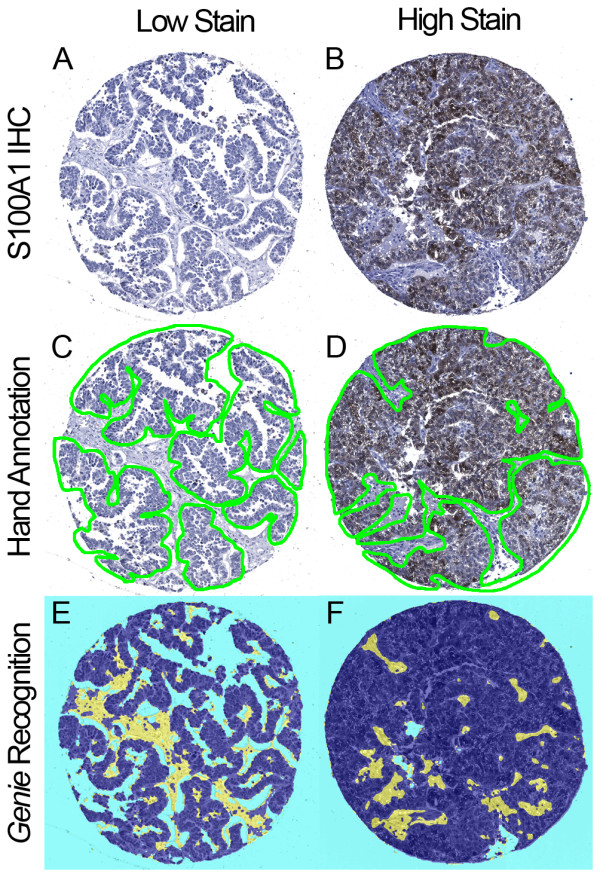
**Manual and automated annotations of ovarian serous carcinoma.** Ovarian serous carcinoma TMA spots immunohistochemically stained for S100A1. Representative lowly and highly stained spots are shown (**A-B**). Image data were processed by both manual pathologist-supervised hand annotations and automated Genie Histology Pattern Recognition software. Digital hand annotations are presented as green outlines of carcinoma, excluding stroma and minimizing background and glass (**C-D**). These same TMA spots were classified by Genie as carcinoma (dark blue), stroma (yellow), and glass (light blue) (**E-F**).

Representative TMA spots that had been stained for S100A1 by IHC were used for the analysis in this study are shown in Figure 1A,B. Examples of pathologist-directed, technician hand-annotation of areas of carcinoma, used in subsequent training and analysis, are shown in Figure 1C,D. The Genie Training Set algorithm was optimized and validated on TMA 1, a process that required one hour of pathologist time in addition to ten hours of technician time. After optimization, the Genie Classifier algorithm was then run on all spots from TMAs 1-4 to classify areas of carcinoma, stroma and glass (Figure 1E,F). For both hand annotated and Genie classified carcinomatous areas, the Color Deconvolution algorithm was run to obtain %Pos and OD*%Pos data for DAB staining. The process of generating final data, which involved image quality control - for example to exclude damaged TMA spots from analysis - and organizing data output from Color Deconvolution, required an average of 3.5 hours per TMA, or 14 hours in total, of technician time.

**Figure 2:**
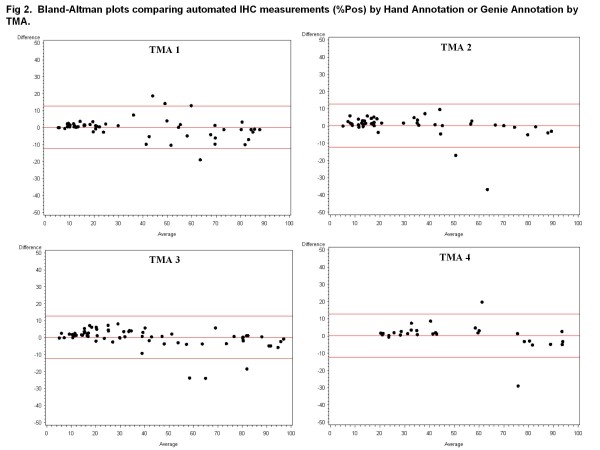
**Bland-Altman plots comparing automated IHC measurements (%Pos) by Hand Annotation or Genie Annotation by TMA.** Bland-Altman difference plots between hand-annotated carcinomatous areas and Genie-annotated carcinomatous areas were generated for %Pos obtained using the Color Deconvolution algorithm. Data are displayed separately for TMA 1 on which the software methods were trained and TMAs 2-4 which were independent data sets. Red lines indicate mean and ± 2*standard deviation.

**Figure 3:**
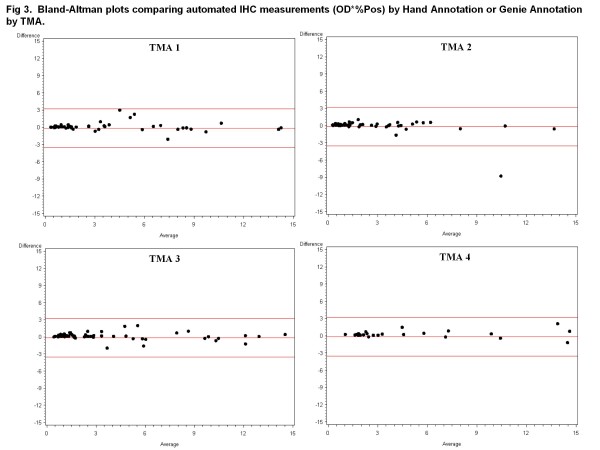
Bland-Altman plots comparing automated IHC measurements (OD*%Pos) by Hand Annotation or Genie Annotation by TMA. Bland-Altman difference plots between hand-annotated carcinomatous areas and Genie-annotated carcinomatous areas were generated for OD*%Pos obtained using the Color Deconvolution algorithm. Data are displayed separately for TMA 1 on which the software methods were trained and TMAs 2-4 which were independent data sets. Red lines indicate mean and ± 2*standard deviation.

There was strong agreement between data resulting from hand-annotation of carcinoma and data obtained after automated Genie classification of carcinoma (Figures 2 and 3). There was stronger agreement between the pathologist hand and automated Genie annotations for the OD*%Pos metric, evidenced by the lower variability in the mean difference in comparison with the %Pos metric.

### Pathologist visual scoring in carcinoma versus Automated IHC measurement in Genie-classified carcinomatous areas

**Figure 4:**
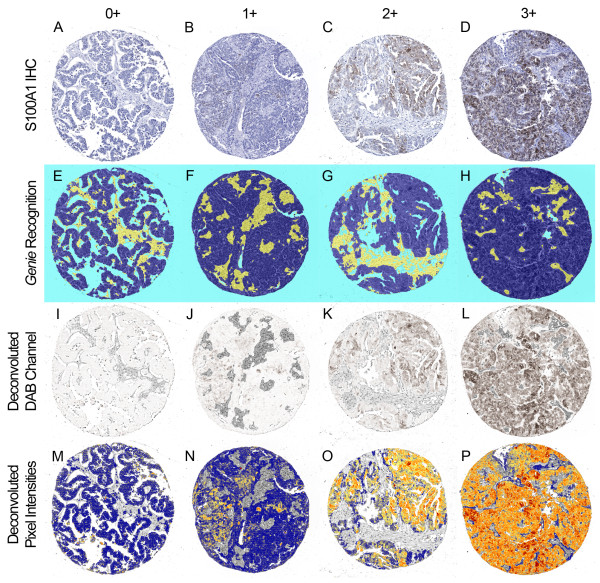
**Representative comparisons of pathologist visual scoring with automated IHC measurement.** Ovarian serous carcinoma TMA spots stained for S100A1 were interpreted by pathologist visual scoring as 0 (no staining), 1 (<10% of carcinoma staining), 2 (10%-50% of carcinoma staining), or 3 (>50% of carcinoma staining). Representative spot for each score is shown as **A-D;** each column shows the identical TMA spot processed by digital methods. Genie Histology Pattern Recognition software classified tissue areas into carcinoma (dark blue), stroma (yellow), or glass (light blue) (**E-H**). Color Deconvolution software individually analyzed DAB staining (deconvolved by its RGB color components; **I-L**), and measured staining intensity only within areas classified as carcinoma. Pseudocolors represent staining intensity in shown as **M-P** (gray = image areas not annotated by Genie as carcinoma and therefore not considered; blue = no staining, yellow = low intensities, orange = medium intensities, and red = high intensities in Genie-annotated carcinomatous areas considered).

**Figure 5:**
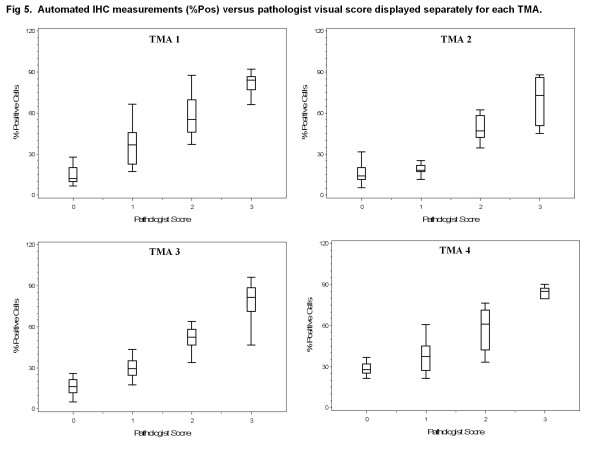
**Automated IHC measurements (%Pos) versus pathologist visual score displayed separately for each TMA.** Box plots of %Pos data generated using Genie Histology Pattern Recognition software and Color Deconvolution software within carcinomatous areas (vertical axes) versus pathologist visual score (horizontal axes). Data are displayed separately for TMA 1 on which the software methods were trained and TMAs 2-4 which were independent data sets.

**Figure 6:**
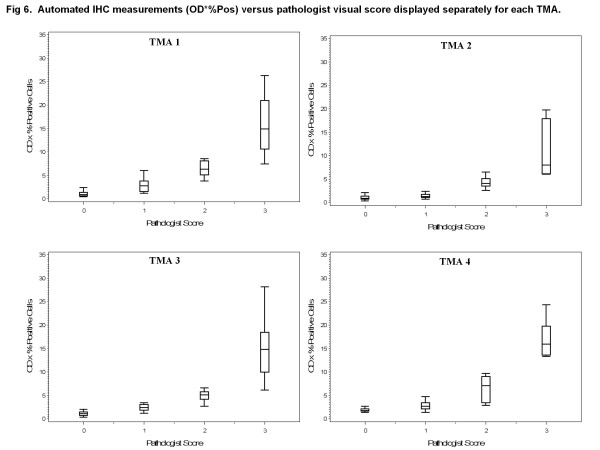
Automated IHC measurements (OD*%Pos) versus pathologist visual score displayed separately for each TMA. Box plots of OD*%Pos data generated using Genie Histology Pattern Recognition software and Color Deconvolution software within carcinomatous areas (vertical axes) versus pathologist visual score (horizontal axes). Data are displayed separately for TMA 1 on which the software methods were trained and TMAs 2-4 which were independent data sets.

Using glass slides, a pathologist scored TMA spots for the percentage of positively stained carcinoma on a scale of 0-3+ as shown in representative spots covering the full scoring range in Figure 4A-D. For the 215 tumors in this study, scoring the TMA spots required 10 hours of pathologist time. In areas classified by Genie as carcinoma (Figure 4E-H), the Color Deconvolution algorithm individually analyzed DAB staining (deconvoluted by its RGB color components; Figure 4I-L) and %Pos and OD*%Pos data were obtained. As in Figure 1E,F, only the areas of carcinoma (pseudocolored as dark blue in Figure 1E,F and Figure 4E-H) were considered; areas of stroma and glass (yellow and light blue, respectively, in Figure 1E-F and Figure 4E-H) did not contribute to the final IHC data. Data representative of OD*%Pos are illustrated as a heatmap in Figure 4M-P (gray = image areas not annotated by Genie as carcinoma and therefore not considered; blue = no staining, yellow = low intensities, orange = medium intensities, and red = high intensities in Genie-annotated carcinomatous areas considered). There was high correlation between pathologist visual scoring and %Pos data obtained using image analysis software for all TMAs, with Spearman correlations of 0.89, 0.78, 0.90 , and 0.90 for TMAs 1, 2, 3, and 4, respectively (all p < 0.0001; box plots of data shown in Figure 5). There was slightly higher correlation between pathologist visual scoring and OD*%Pos data, with Spearman correlations of 0.91, 0.81, 0.90, and 0.91, for TMAs 1, 2, 3, and 4, respectively (all p < 0.0001; box plots shown in Figure 6).

We next compared pathologist visual scoring with combined data (TMAs 1-4) from digital image analysis, revealing high correlation between pathologist visual scoring and %Pos (Spearman correlation 0.88, p < 0.0001) and OD*%Pos (Spearman correlation 0.90, p < 0.0001). There were significant differences in the median values for both metrics (%Pos and OD*%Pos) by pathologist score. Most notably, there were significant differences in computer-derived data corresponding to spots scored by the pathologists as “0” and “1” for both %Pos (p < 0.0001) and OD*%Pos (p < 0.0001).

## Discussion

In this report we have demonstrated that commercially available software algorithms to classify disease-relevant tissue areas (Genie Histology Pattern Recognition) and quantify IHC staining within those areas (Color Deconvolution) effectively replicated IHC data produced by manual classification of image areas and pathologist visual scoring for S100A1 in ovarian serous carcinoma. Other software algorithms also provide data highly correlated with pathologist scores, e.g., human epidermal growth factor receptor 2 (HER2) [28-34], estrogen receptor [35-39] and progesterone receptor [37-39] in breast cancer, DNA mismatch repair proteins in esophageal cancer [40], and epidermal growth factor receptor signaling molecules in colon cancer [41], among other biomarkers.

In this report, we provide important additional information regarding comparisons between digital data based solely on IHC-positive area (%Pos) and data combining area and staining intensity (OD*%Pos). The OD*%Pos metric provided better visual correlation between hand-annotated areas and Genie-annotated areas (Figure 4). Further, the OD*%Pos metric provided slightly higher correlation between digital IHC data and pathologist visual scoring. Of note, the study pathologist (S.E.P.) scored TMA spots for this study based on IHC-stained area as described in the Materials and Methods section, rather than by using a method such as HSCORE, which summated the percentage of area stained at each intensity level multiplied by the weighted intensity (e.g., 1, 2, or 3) [12]. Thus, it is unclear from our data why OD*%Pos performed somewhat better than %Pos. We speculate that, since the human eye is more sensitive to higher intensity IHC staining [22], the estimation by eye of area IHC-stained likely inherently encompasses a component of staining intensity.

We additionally provide information regarding time conservation for pathologists using digital imaging methods for obtaining IHC data. While acknowledging that generating the automated IHC measurements within Genie-classified areas of carcinoma required 24 hours of technician time, 10-fold less pathologist time was required versus visual examination of each spot on TMAs 1-4. Greater efficiencies in the use of pathologists’ time are needed as pathologists are experiencing increasing demands on their time due to higher clinical practice volumes, greater complexity of testing, and industry-wide shortages in available employees [42]. Although we did not measure pathologist time on a per-TMA spot basis in this study, a previous study indicates that per-spot time required for pathologist visual scoring of TMAs markedly increases as the number of spots to be analyzed increases [43]. Although limited data are available to assess pathologist fatigue on data quality, fatigue is postulated as a potential source of error in visual interpretation of IHC stained tissue sections [17]. To the contrary, automated analysis is objective and temporally linear regardless of the number of spots analyzed [43].

Although IHC biomarker studies widely use pathologist visual scoring, automated IHC measurement offers several additional advantages. First, pathologist visual scoring is fraught with data quality problems. The human eye is least accurate at detecting differences under conditions of weak staining at which IHC is most linearly related to target antigen concentration [22]. Consequently, regions of negative and high-positive intensities may be overcalled leading to artificially-produced bimodal score distributions [23]. While pathologist-derived data have good to excellent intra- and inter-observer reproducibility [18-20], estimation of percentages of areas stained has only poor to good reproducibility [19]. Digital methods may provide more reliable data. For example, automated HER2 IHC measurements are more comparable to consensus visual scores by multiple expert pathologists, and to HER2 gene amplification data, than are individual pathologists’ subjective visual scores [44]. Since consensus scoring by experts is impractical in routine practice, automated IHC measurement may provide a means to improve IHC data quality. Intra- and inter-observer agreement is improved by providing pathologists with computer-aided IHC measurements during the visual scoring process [20,45]. Software algorithms such as Genie and Color Deconvolution may be “locked” such that all subsequent images are analyzed using the same parameters. Second, the automated methods demonstrated in this report also produced continuous variable data. Recent studies indicate that continuous variable data may allow identification of IHC cut-points of prognostic relevance that are either undetected [46] or are less statistically significant [23,34,47] by visual scoring. Third, digital methods support multigene expression studies at the protein level. Methods exist to multiplex IHC using immunofluorescence [48], destaining and restaining protocols [49], multiple chromagens [50,51], and combining data from adjacent tissue sections [52,53]. Based on these and other studies, automated methods will likely become standard clinical practice.

## Conclusions

This study demonstrated the effectiveness of optimized histology pattern recognition and automated IHC measurement algorithms to reproduce manual annotations and visual evaluation by a pathologist. This approach used TMAs in which tissue cores were obtained under the direction of a pathologist from areas containing exclusively tumor. A limited number of tissue cores adequately represent protein expression in tumor specimens [15,16]. Nevertheless, methods of quality control are required in final data analysis to exclude tissue areas with artifacts such as tissue folds, and tissue regions not of interest such as admixed benign tissue elements in the analysis of carcinoma. It is important to note that we have found, in data not shown, that each combination of tissue type and IHC stain requires separate Genie optimization.

## Abbreviations

CI = Confidence interval; DAB 3 = 3-diaminobenzidine; FFPE = Formalin-fixed paraffin-embedded; HER2 = Human epidermal growth factor receptor 2; IHC = Immunohistochemistry; OD*%Pos = Product of the staining intensity multiplied by the percentage of carcinoma with immunohistochemical staining; %Pos percentage of carcinoma with immunohistochemical staining; TMA = Tissue microarray.

## Competing interest

The authors declare no conflict of interest.

## Authors' contributions

AER participated in study design, execution, analysis and interpretation of data, and drafting the manuscript. ATJ participated in study design and execution and analysis of data. RIV participated in study design, analysis and interpretation of data, and drafting the manuscript. SEP participated in execution of the study, interpretation of data, and reviewing the manuscript. JH assisted in execution of the study. APNS participated in execution of the study and reviewing the manuscript. GJM assisted in drafting the manuscript. SCS conceived of the study design, participated in data analysis and interpretation, and in drafting the manuscript. All authors read and approved the final manuscript.
